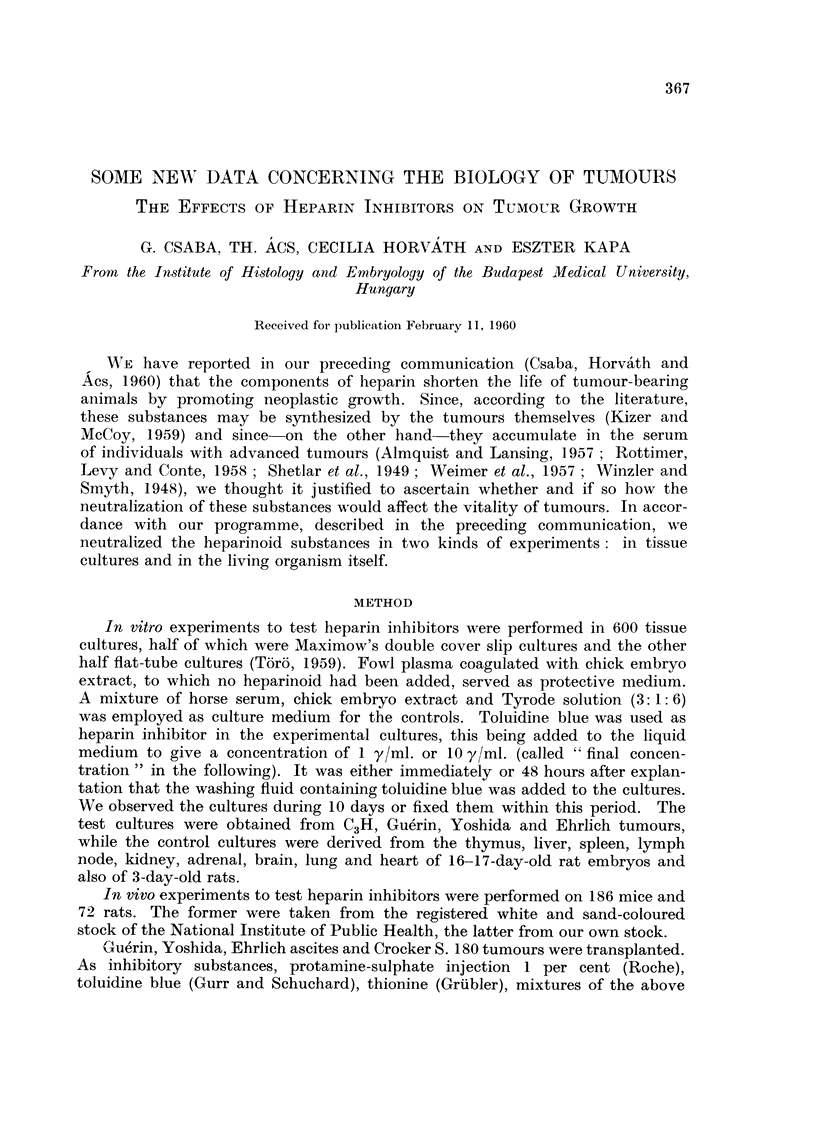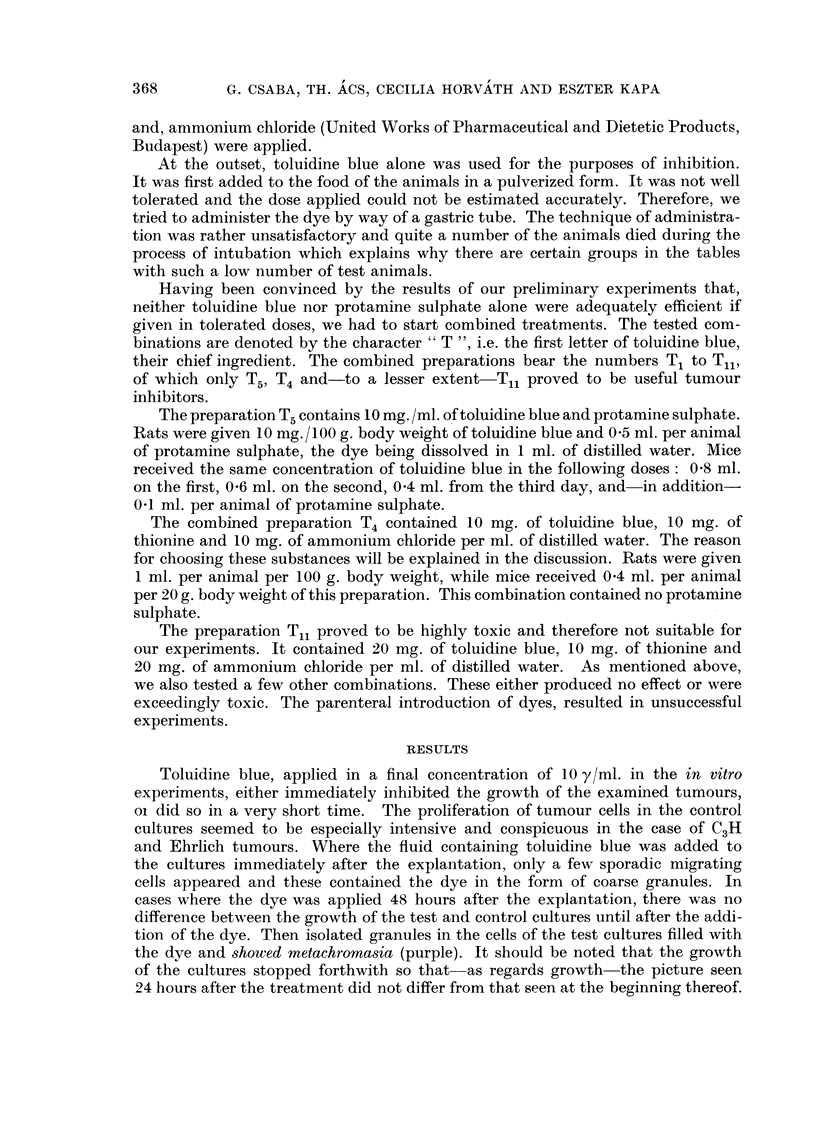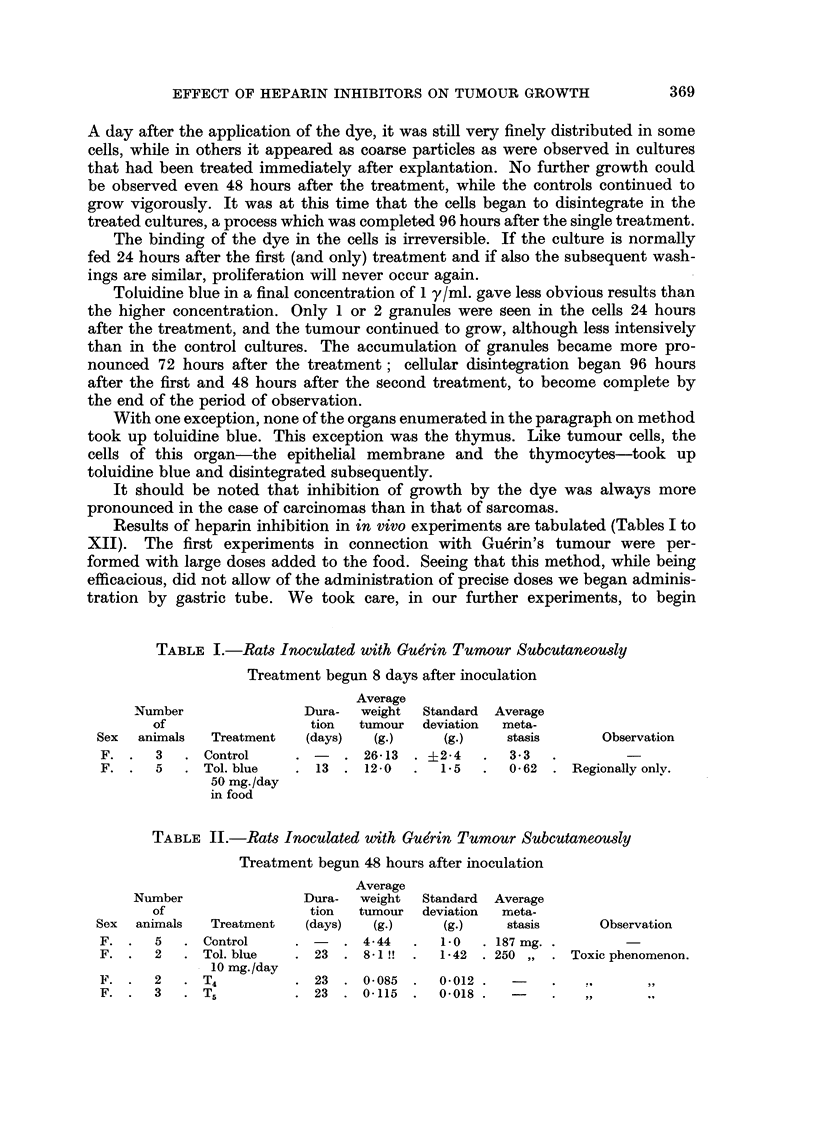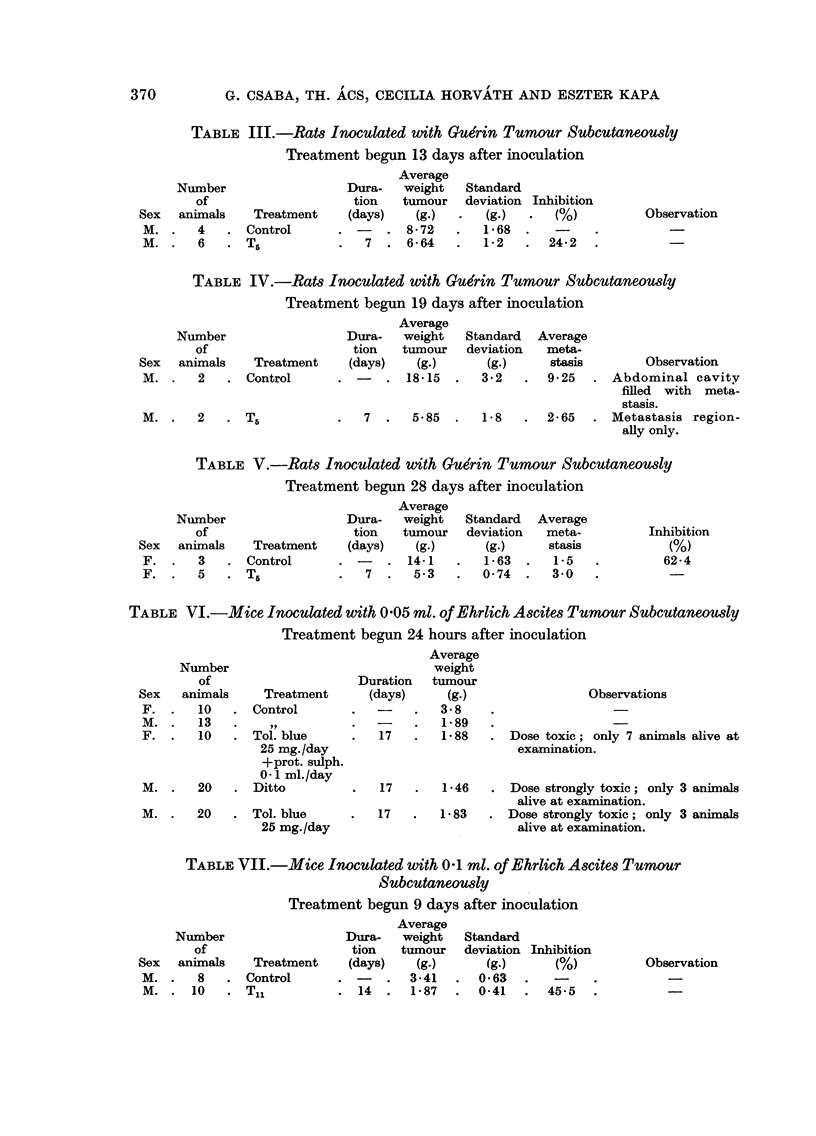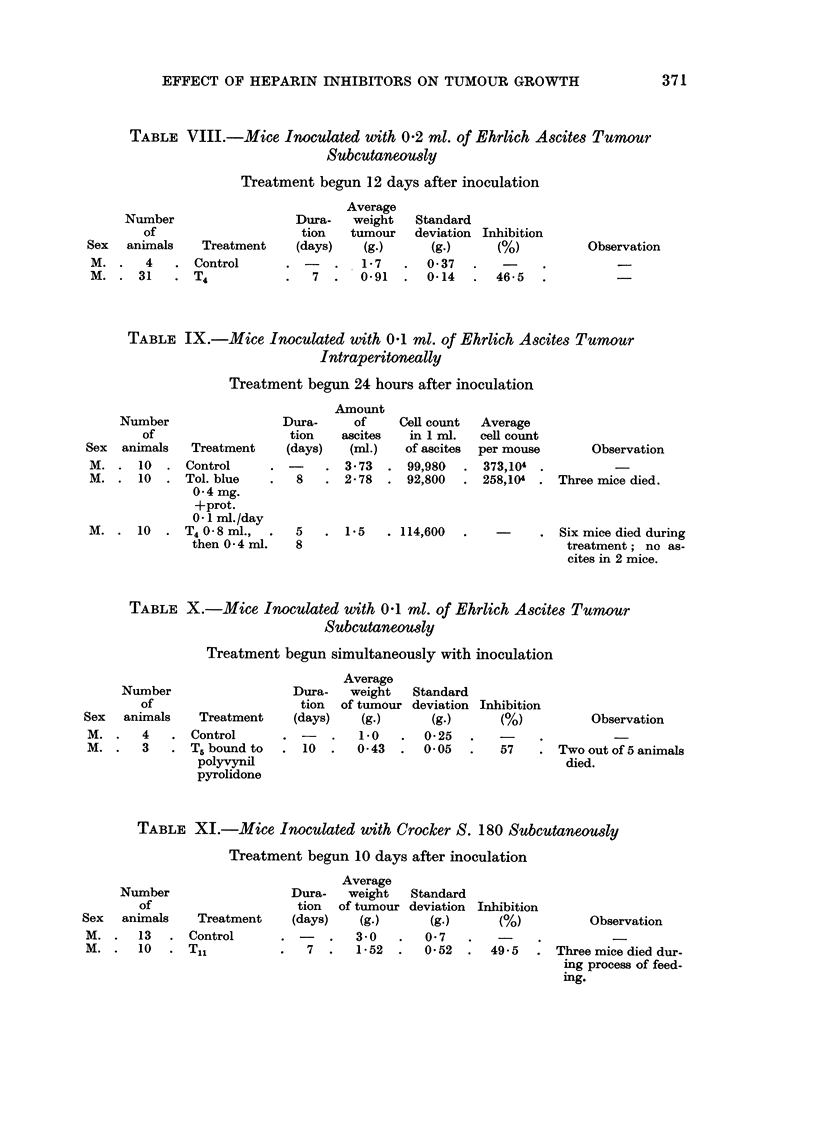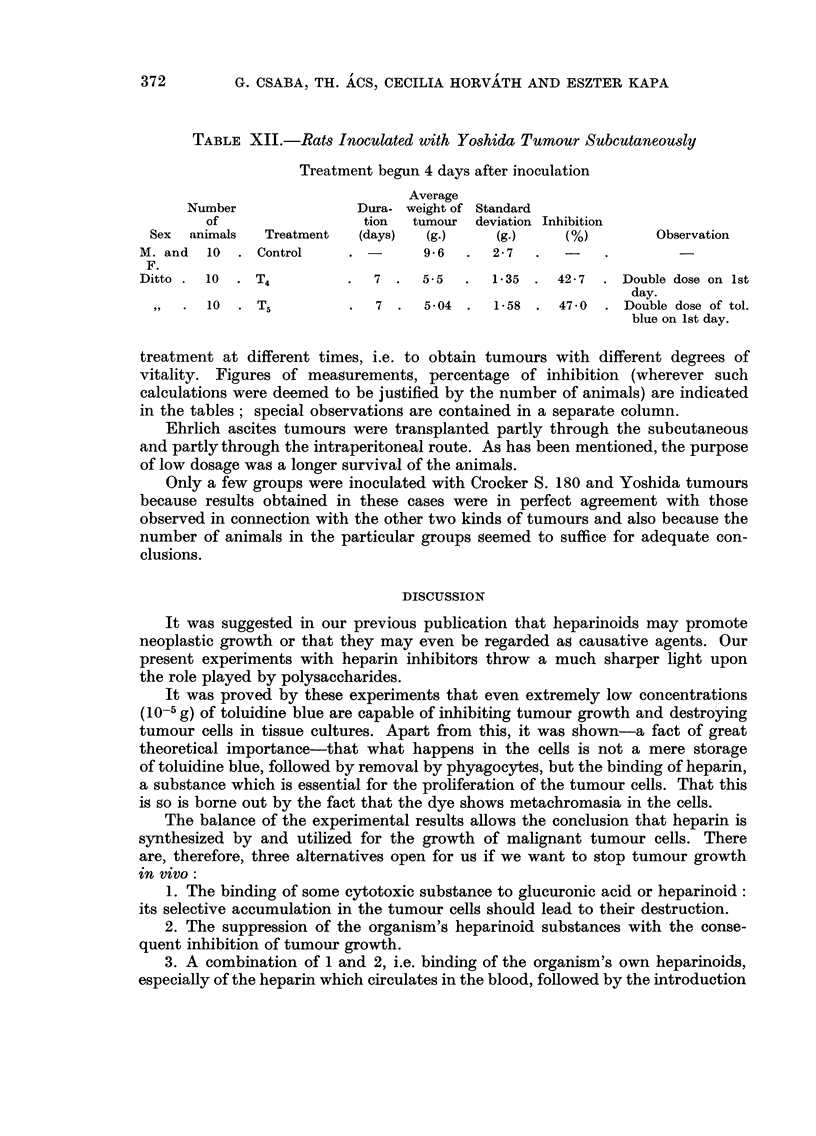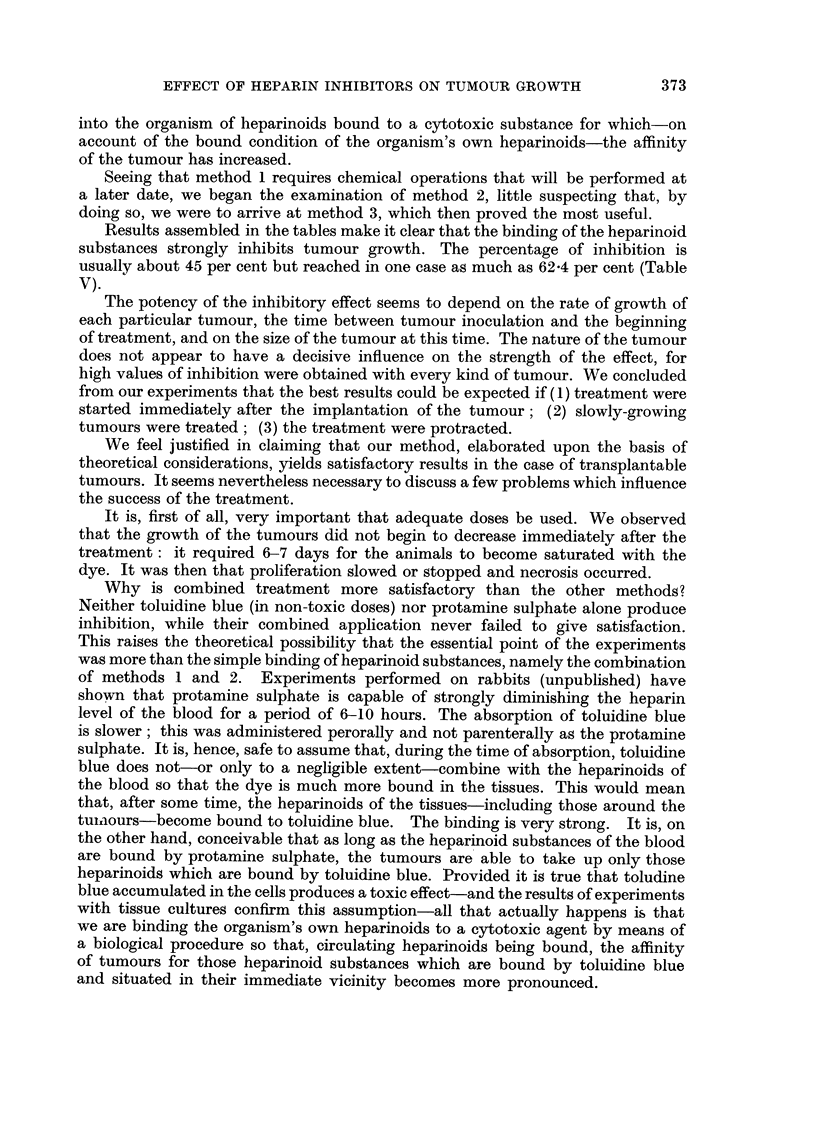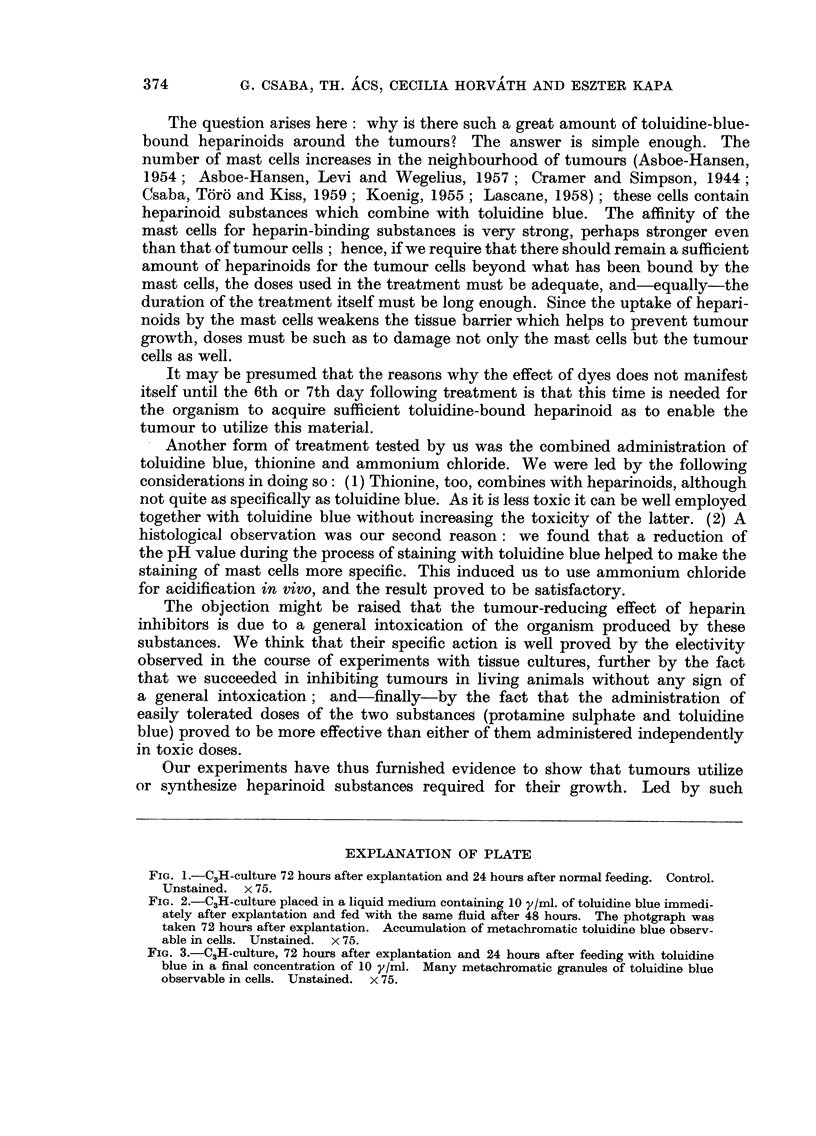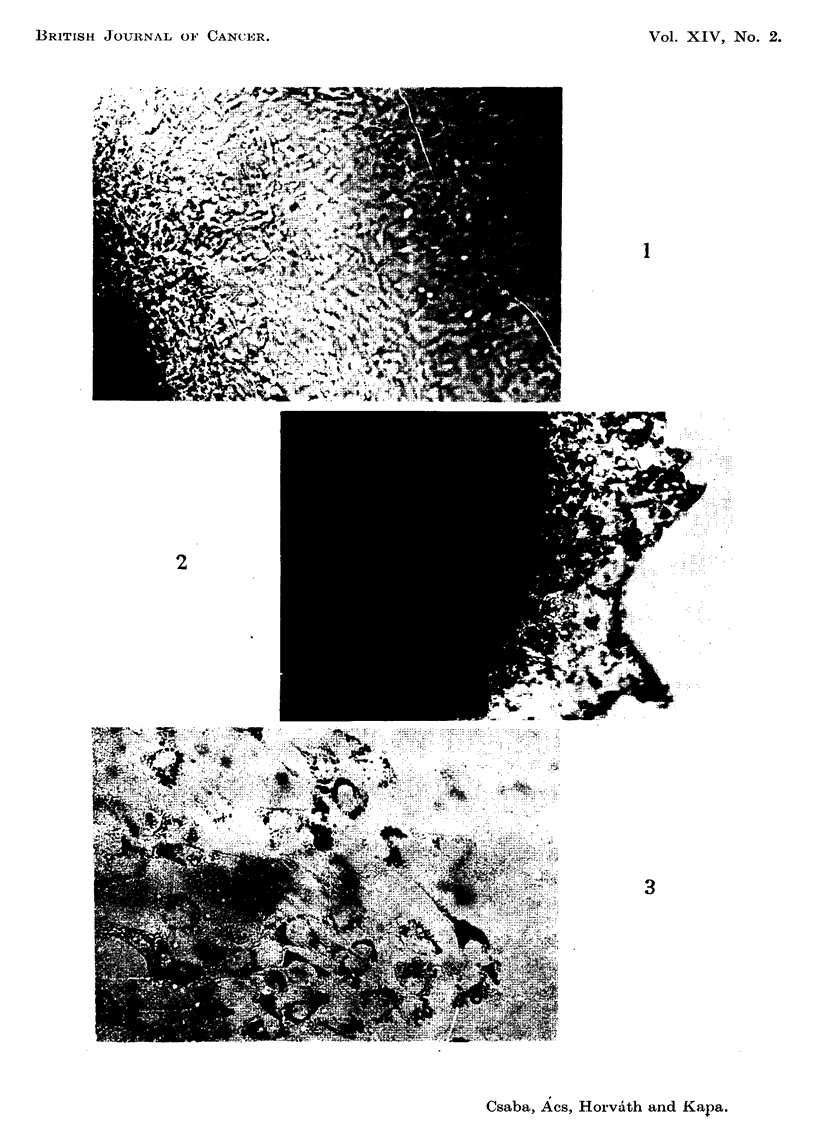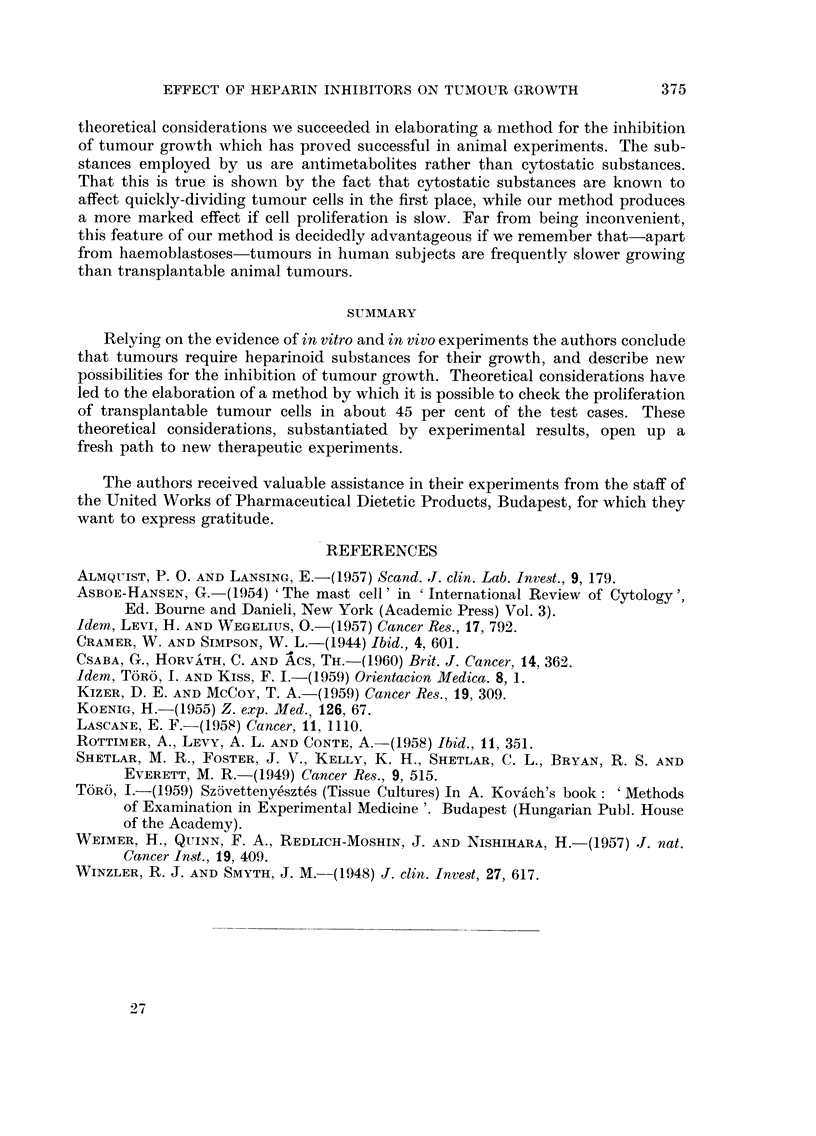# Some New Data concerning the Biology of Tumours

**DOI:** 10.1038/bjc.1960.43

**Published:** 1960-06

**Authors:** G. Csaba, Th. Ács, Cecilia Horváth, Eszter Kapa

## Abstract

**Images:**


### SOME NEW DATA CONCERNING THE BIOLOGY OF TUMOURS

### THE EFFECTS OF HEPARIN INHIBITORS ON TUMOUR GROWTH

G. CSABA, TH. ÁCS, CECILIA HORVÁTH AND ESZTER KAPA

*From the Institute of Histology and Embryology of the Budapest Medical University, Hungary*

Received for publication February 11, 1960

WE have reported in our preceding communication (Csaba, Horváth and Ács, 1960) that the components of heparin shorten the life of tumour-bearing animals by promoting neoplastic growth. Since, according to the literature, these substances may be synthesized by the tumours themselves (Kizer and McCoy, 1959) and since—on the other hand—they accumulate in the serum of individuals with advanced tumours (Almquist and Lansing, 1957 ; Rottimer, Levy and Conte, 1958 ; Shetlar *et al.*, 1949 ; Weimer *et al.*, 1957 ; Winzler and Smyth, 1948), we thought it justified to ascertain whether and if so how the neutralization of these substances would affect the vitality of tumours. In accordance with our programme, described in the preceding communication, we neutralized the heparinoid substances in two kinds of experiments : in tissue cultures and in the living organism itself.

### METHOD

*In vitro* experiments to test heparin inhibitors were performed in 600 tissue cultures, half of which were Maximow's double cover slip cultures and the other half flat-tube cultures (Törö, 1959). Fowl plasma coagulated with chick embryo extract, to which no heparinoid had been added, served as protective medium. A mixture of horse serum, chick embryo extract and Tyrode solution (3 : 1 : 6) was employed as culture medium for the controls. Toluidine blue was used as heparin inhibitor in the experimental cultures, this being added to the liquid medium to give a concentration of 1 $\gamma$/ml. or 10 $\gamma$/ml. (called " final concentration " in the following). It was either immediately or 48 hours after explantation that the washing fluid containing toluidine blue was added to the cultures. We observed the cultures during 10 days or fixed them within this period. The test cultures were obtained from $C_3H$, Guérin, Yoshida and Ehrlich tumours, while the control cultures were derived from the thymus, liver, spleen, lymph node, kidney, adrenal, brain, lung and heart of 16–17-day-old rat embryos and also of 3-day-old rats.

*In vivo* experiments to test heparin inhibitors were performed on 186 mice and 72 rats. The former were taken from the registered white and sand-coloured stock of the National Institute of Public Health, the latter from our own stock.

Guérin, Yoshida, Ehrlich ascites and Crocker S. 180 tumours were transplanted. As inhibitory substances, protamine-sulphate injection 1 per cent (Roche), toluidine blue (Gurr and Schuchard), thionine (Grübler), mixtures of the above and, ammonium chloride (United Works of Pharmaceutical and Dietetic Products, Budapest) were applied.

At the outset, toluidine blue alone was used for the purposes of inhibition. It was first added to the food of the animals in a pulverized form. It was not well tolerated and the dose applied could not be estimated accurately. Therefore, we tried to administer the dye by way of a gastric tube. The technique of administration was rather unsatisfactory and quite a number of the animals died during the process of intubation which explains why there are certain groups in the tables with such a low number of test animals.

Having been convinced by the results of our preliminary experiments that, neither toluidine blue nor protamine sulphate alone were adequately efficient if given in tolerated doses, we had to start combined treatments. The tested combinations are denoted by the character " T ", i.e. the first letter of toluidine blue, their chief ingredient. The combined preparations bear the numbers $T_1$ to $T_{11}$, of which only $T_5$, $T_4$ and—to a lesser extent—$T_{11}$ proved to be useful tumour inhibitors.

The preparation $T_5$ contains 10 mg./ml. of toluidine blue and protamine sulphate. Rats were given 10 mg./100 g. body weight of toluidine blue and 0·5 ml. per animal of protamine sulphate, the dye being dissolved in 1 ml. of distilled water. Mice received the same concentration of toluidine blue in the following doses : 0·8 ml. on the first, 0·6 ml. on the second, 0·4 ml. from the third day, and—in addition—0·1 ml. per animal of protamine sulphate.

The combined preparation $T_4$ contained 10 mg. of toluidine blue, 10 mg. of thionine and 10 mg. of ammonium chloride per ml. of distilled water. The reason for choosing these substances will be explained in the discussion. Rats were given 1 ml. per animal per 100 g. body weight, while mice received 0·4 ml. per animal per 20 g. body weight of this preparation. This combination contained no protamine sulphate.

The preparation $T_{11}$ proved to be highly toxic and therefore not suitable for our experiments. It contained 20 mg. of toluidine blue, 10 mg. of thionine and 20 mg. of ammonium chloride per ml. of distilled water. As mentioned above, we also tested a few other combinations. These either produced no effect or were exceedingly toxic. The parenteral introduction of dyes, resulted in unsuccessful experiments.

### RESULTS

Toluidine blue, applied in a final concentration of 10 $\gamma$/ml. in the *in vitro* experiments, either immediately inhibited the growth of the examined tumours, oı did so in a very short time. The proliferation of tumour cells in the control cultures seemed to be especially intensive and conspicuous in the case of $C_3H$ and Ehrlich tumours. Where the fluid containing toluidine blue was added to the cultures immediately after the explantation, only a few sporadic migrating cells appeared and these contained the dye in the form of coarse granules. In cases where the dye was applied 48 hours after the explantation, there was no difference between the growth of the test and control cultures until after the addition of the dye. Then isolated granules in the cells of the test cultures filled with the dye and *showed metachromasia* (purple). It should be noted that the growth of the cultures stopped forthwith so that—as regards growth—the picture seen 24 hours after the treatment did not differ from that seen at the beginning thereof.

A day after the application of the dye, it was still very finely distributed in some cells, while in others it appeared as coarse particles as were observed in cultures that had been treated immediately after explantation. No further growth could be observed even 48 hours after the treatment, while the controls continued to grow vigorously. It was at this time that the cells began to disintegrate in the treated cultures, a process which was completed 96 hours after the single treatment.

The binding of the dye in the cells is irreversible. If the culture is normally fed 24 hours after the first (and only) treatment and if also the subsequent washings are similar, proliferation will never occur again.

Toluidine blue in a final concentration of 1 $\gamma$/ml. gave less obvious results than the higher concentration. Only 1 or 2 granules were seen in the cells 24 hours after the treatment, and the tumour continued to grow, although less intensively than in the control cultures. The accumulation of granules became more pronounced 72 hours after the treatment; cellular disintegration began 96 hours after the first and 48 hours after the second treatment, to become complete by the end of the period of observation.

With one exception, none of the organs enumerated in the paragraph on method took up toluidine blue. This exception was the thymus. Like tumour cells, the cells of this organ—the epithelial membrane and the thymocytes—took up toluidine blue and disintegrated subsequently.

It should be noted that inhibition of growth by the dye was always more pronounced in the case of carcinomas than in that of sarcomas.

Results of heparin inhibition in *in vivo* experiments are tabulated (Tables I to XII). The first experiments in connection with Guérin's tumour were performed with large doses added to the food. Seeing that this method, while being efficacious, did not allow of the administration of precise doses we began administration by gastric tube. We took care, in our further experiments, to begin

TABLE I.—*Rats Inoculated with Guérin Tumour Subcutaneously*

Treatment begun 8 days after inoculation

| Sex | Number of animals | Treatment | Duration (days) | Average weight tumour (g.) | Standard deviation (g.) | Average metastasis | Observation |
|---|---|---|---|---|---|---|---|
| F. | 3 | Control | — | 26·13 | ±2·4 | 3·3 | — |
| F. | 5 | Tol. blue 50 mg./day in food | 13 | 12·0 | 1·5 | 0·62 | Regionally only. |

TABLE II.—*Rats Inoculated with Guérin Tumour Subcutaneously*

Treatment begun 48 hours after inoculation

| Sex | Number of animals | Treatment | Duration (days) | Average weight tumour (g.) | Standard deviation (g.) | Average metastasis | Observation |
|---|---|---|---|---|---|---|---|
| F. | 5 | Control | — | 4·44 | 1·0 | 187 mg. | — |
| F. | 2 | Tol. blue 10 mg./day | 23 | 8·1 !! | 1·42 | 250 ,, | Toxic phenomenon. |
| F. | 2 | $T_4$ | 23 | 0·085 | 0·012 | — | ,, ,, |
| F. | 3 | $T_5$ | 23 | 0·115 | 0·018 | — | ,, ,, |

TABLE III.—*Rats Inoculated with Guérin Tumour Subcutaneously*

Treatment begun 13 days after inoculation

| Sex | Number of animals | Treatment | Duration (days) | Average weight tumour (g.) | Standard deviation (g.) | Inhibition (%) | Observation |
|---|---|---|---|---|---|---|---|
| M. | 4 | Control | — | 8·72 | 1·68 | — | — |
| M. | 6 | $T_5$ | 7 | 6·64 | 1·2 | 24·2 | — |

TABLE IV.—*Rats Inoculated with Guérin Tumour Subcutaneously*

Treatment begun 19 days after inoculation

| Sex | Number of animals | Treatment | Duration (days) | Average weight tumour (g.) | Standard deviation (g.) | Average metastasis | Observation |
|---|---|---|---|---|---|---|---|
| M. | 2 | Control | — | 18·15 | 3·2 | 9·25 | Abdominal cavity filled with metastasis. |
| M. | 2 | $T_5$ | 7 | 5·85 | 1·8 | 2·65 | Metastasis regionally only. |

TABLE V.—*Rats Inoculated with Guérin Tumour Subcutaneously*

Treatment begun 28 days after inoculation

| Sex | Number of animals | Treatment | Duration (days) | Average weight tumour (g.) | Standard deviation (g.) | Average metastasis | Inhibition (%) |
|---|---|---|---|---|---|---|---|
| F. | 3 | Control | — | 14·1 | 1·63 | 1·5 | 62·4 |
| F. | 5 | $T_5$ | 7 | 5·3 | 0·74 | 3·0 | — |

TABLE VI.—*Mice Inoculated with* 0·05 *ml. of Ehrlich Ascites Tumour Subcutaneously*

Treatment begun 24 hours after inoculation

| Sex | Number of animals | Treatment | Duration (days) | Average weight tumour (g.) | Observations |
|---|---|---|---|---|---|
| F. | 10 | Control | — | 3·8 | — |
| M. | 13 | ,, | — | 1·89 | — |
| F. | 10 | Tol. blue 25 mg./day +prot. sulph. 0·1 ml./day | 17 | 1·88 | Dose toxic; only 7 animals alive at examination. |
| M. | 20 | Ditto | 17 | 1·46 | Dose strongly toxic; only 3 animals alive at examination. |
| M. | 20 | Tol. blue 25 mg./day | 17 | 1·83 | Dose strongly toxic; only 3 animals alive at examination. |

TABLE VII.—*Mice Inoculated with* 0·1 *ml. of Ehrlich Ascites Tumour Subcutaneously*

Treatment begun 9 days after inoculation

| Sex | Number of animals | Treatment | Duration (days) | Average weight tumour (g.) | Standard deviation (g.) | Inhibition (%) | Observation |
|---|---|---|---|---|---|---|---|
| M. | 8 | Control | — | 3·41 | 0·63 | — | — |
| M. | 10 | $T_{11}$ | 14 | 1·87 | 0·41 | 45·5 | — |

TABLE VIII.—*Mice Inoculated with* 0·2 *ml. of Ehrlich Ascites Tumour Subcutaneously*

Treatment begun 12 days after inoculation

| Sex | Number of animals | Treatment | Duration (days) | Average weight tumour (g.) | Standard deviation (g.) | Inhibition (%) | Observation |
|---|---|---|---|---|---|---|---|
| M. | 4 | Control | — | 1·7 | 0·37 | — | — |
| M. | 31 | $T_4$ | 7 | 0·91 | 0·14 | 46·5 | — |

TABLE IX.—*Mice Inoculated with* 0·1 *ml. of Ehrlich Ascites Tumour Intraperitoneally*

Treatment begun 24 hours after inoculation

| Sex | Number of animals | Treatment | Duration (days) | Amount of ascites (ml.) | Cell count in 1 ml. of ascites | Average cell count per mouse | Observation |
|---|---|---|---|---|---|---|---|
| M. | 10 | Control | — | 3·73 | 99,980 | 373,10[4] | — |
| M. | 10 | Tol. blue 0·4 mg. +prot. 0·1 ml./day | 8 | 2·78 | 92,800 | 258,10[4] | Three mice died. |
| M. | 10 | $T_4$ 0·8 ml., then 0·4 ml. | 5<br>8 | 1·5 | 114,600 | — | Six mice died during treatment; no ascites in 2 mice. |

TABLE X.—*Mice Inoculated with* 0·1 *ml. of Ehrlich Ascites Tumour Subcutaneously*

Treatment begun simultaneously with inoculation

| Sex | Number of animals | Treatment | Duration (days) | Average weight of tumour (g.) | Standard deviation (g.) | Inhibition (%) | Observation |
|---|---|---|---|---|---|---|---|
| M. | 4 | Control | — | 1·0 | 0·25 | — | — |
| M. | 3 | $T_5$ bound to polyvynil pyrolidone | 10 | 0·43 | 0·05 | 57 | Two out of 5 animals died. |

TABLE XI.—*Mice Inoculated with Crocker S.* 180 *Subcutaneously*

Treatment begun 10 days after inoculation

| Sex | Number of animals | Treatment | Duration (days) | Average weight of tumour (g.) | Standard deviation (g.) | Inhibition (%) | Observation |
|---|---|---|---|---|---|---|---|
| M. | 13 | Control | — | 3·0 | 0·7 | — | — |
| M. | 10 | $T_{11}$ | 7 | 1·52 | 0·52 | 49·5 | Three mice died during process of feeding. |

TABLE XII.—*Rats Inoculated with Yoshida Tumour Subcutaneously*

Treatment begun 4 days after inoculation

| Sex | Number of animals | Treatment | Duration (days) | Average weight of tumour (g.) | Standard deviation (g.) | Inhibition (%) | Observation |
|---|---|---|---|---|---|---|---|
| M. and F. | 10 | Control | — | 9·6 | 2·7 | — | — |
| Ditto | 10 | $T_4$ | 7 | 5·5 | 1·35 | 42·7 | Double dose on 1st day. |
| ,, | 10 | $T_5$ | 7 | 5·04 | 1·58 | 47·0 | Double dose of tol. blue on 1st day. |

treatment at different times, i.e. to obtain tumours with different degrees of vitality. Figures of measurements, percentage of inhibition (wherever such calculations were deemed to be justified by the number of animals) are indicated in the tables ; special observations are contained in a separate column.

Ehrlich ascites tumours were transplanted partly through the subcutaneous and partly through the intraperitoneal route. As has been mentioned, the purpose of low dosage was a longer survival of the animals.

Only a few groups were inoculated with Crocker S. 180 and Yoshida tumours because results obtained in these cases were in perfect agreement with those observed in connection with the other two kinds of tumours and also because the number of animals in the particular groups seemed to suffice for adequate conclusions.

### DISCUSSION

It was suggested in our previous publication that heparinoids may promote neoplastic growth or that they may even be regarded as causative agents. Our present experiments with heparin inhibitors throw a much sharper light upon the role played by polysaccharides.

It was proved by these experiments that even extremely low concentrations ($10^{-5}$ g) of toluidine blue are capable of inhibiting tumour growth and destroying tumour cells in tissue cultures. Apart from this, it was shown—a fact of great theoretical importance—that what happens in the cells is not a mere storage of toluidine blue, followed by removal by phyagocytes, but the binding of heparin, a substance which is essential for the proliferation of the tumour cells. That this is so is borne out by the fact that the dye shows metachromasia in the cells.

The balance of the experimental results allows the conclusion that heparin is synthesized by and utilized for the growth of malignant tumour cells. There are, therefore, three alternatives open for us if we want to stop tumour growth *in vivo* :

1. The binding of some cytotoxic substance to glucuronic acid or heparinoid : its selective accumulation in the tumour cells should lead to their destruction.

2. The suppression of the organism's heparinoid substances with the consequent inhibition of tumour growth.

3. A combination of 1 and 2, i.e. binding of the organism's own heparinoids, especially of the heparin which circulates in the blood, followed by the introduction into the organism of heparinoids bound to a cytotoxic substance for which—on account of the bound condition of the organism's own heparinoids—the affinity of the tumour has increased.

Seeing that method 1 requires chemical operations that will be performed at a later date, we began the examination of method 2, little suspecting that, by doing so, we were to arrive at method 3, which then proved the most useful.

Results assembled in the tables make it clear that the binding of the heparinoid substances strongly inhibits tumour growth. The percentage of inhibition is usually about 45 per cent but reached in one case as much as 62·4 per cent (Table V).

The potency of the inhibitory effect seems to depend on the rate of growth of each particular tumour, the time between tumour inoculation and the beginning of treatment, and on the size of the tumour at this time. The nature of the tumour does not appear to have a decisive influence on the strength of the effect, for high values of inhibition were obtained with every kind of tumour. We concluded from our experiments that the best results could be expected if (1) treatment were started immediately after the implantation of the tumour ; (2) slowly-growing tumours were treated ; (3) the treatment were protracted.

We feel justified in claiming that our method, elaborated upon the basis of theoretical considerations, yields satisfactory results in the case of transplantable tumours. It seems nevertheless necessary to discuss a few problems which influence the success of the treatment.

It is, first of all, very important that adequate doses be used. We observed that the growth of the tumours did not begin to decrease immediately after the treatment : it required 6–7 days for the animals to become saturated with the dye. It was then that proliferation slowed or stopped and necrosis occurred.

Why is combined treatment more satisfactory than the other methods? Neither toluidine blue (in non-toxic doses) nor protamine sulphate alone produce inhibition, while their combined application never failed to give satisfaction. This raises the theoretical possibility that the essential point of the experiments was more than the simple binding of heparinoid substances, namely the combination of methods 1 and 2. Experiments performed on rabbits (unpublished) have shown that protamine sulphate is capable of strongly diminishing the heparin level of the blood for a period of 6–10 hours. The absorption of toluidine blue is slower ; this was administered perorally and not parenterally as the protamine sulphate. It is, hence, safe to assume that, during the time of absorption, toluidine blue does not—or only to a negligible extent—combine with the heparinoids of the blood so that the dye is much more bound in the tissues. This would mean that, after some time, the heparinoids of the tissues—including those around the tumours—become bound to toluidine blue. The binding is very strong. It is, on the other hand, conceivable that as long as the heparinoid substances of the blood are bound by protamine sulphate, the tumours are able to take up only those heparinoids which are bound by toluidine blue. Provided it is true that toludine blue accumulated in the cells produces a toxic effect—and the results of experiments with tissue cultures confirm this assumption—all that actually happens is that we are binding the organism's own heparinoids to a cytotoxic agent by means of a biological procedure so that, circulating heparinoids being bound, the affinity of tumours for those heparinoid substances which are bound by toluidine blue and situated in their immediate vicinity becomes more pronounced.

The question arises here : why is there such a great amount of toluidine-blue-bound heparinoids around the tumours? The answer is simple enough. The number of mast cells increases in the neighbourhood of tumours (Asboe-Hansen, 1954 ; Asboe-Hansen, Levi and Wegelius, 1957 ; Cramer and Simpson, 1944 ; Csaba, Törö and Kiss, 1959 ; Koenig, 1955 ; Lascane, 1958) ; these cells contain heparinoid substances which combine with toluidine blue. The affinity of the mast cells for heparin-binding substances is very strong, perhaps stronger even than that of tumour cells ; hence, if we require that there should remain a sufficient amount of heparinoids for the tumour cells beyond what has been bound by the mast cells, the doses used in the treatment must be adequate, and—equally—the duration of the treatment itself must be long enough. Since the uptake of heparinoids by the mast cells weakens the tissue barrier which helps to prevent tumour growth, doses must be such as to damage not only the mast cells but the tumour cells as well.

It may be presumed that the reasons why the effect of dyes does not manifest itself until the 6th or 7th day following treatment is that this time is needed for the organism to acquire sufficient toluidine-bound heparinoid as to enable the tumour to utilize this material.

Another form of treatment tested by us was the combined administration of toluidine blue, thionine and ammonium chloride. We were led by the following considerations in doing so : (1) Thionine, too, combines with heparinoids, although not quite as specifically as toluidine blue. As it is less toxic it can be well employed together with toluidine blue without increasing the toxicity of the latter. (2) A histological observation was our second reason : we found that a reduction of the pH value during the process of staining with toluidine blue helped to make the staining of mast cells more specific. This induced us to use ammonium chloride for acidification *in vivo*, and the result proved to be satisfactory.

The objection might be raised that the tumour-reducing effect of heparin inhibitors is due to a general intoxication of the organism produced by these substances. We think that their specific action is well proved by the electivity observed in the course of experiments with tissue cultures, further by the fact that we succeeded in inhibiting tumours in living animals without any sign of a general intoxication ; and—finally—by the fact that the administration of easily tolerated doses of the two substances (protamine sulphate and toluidine blue) proved to be more effective than either of them administered independently in toxic doses.

Our experiments have thus furnished evidence to show that tumours utilize or synthesize heparinoid substances required for their growth. Led by such

---

EXPLANATION OF PLATE

FIG. 1.—$C_3H$-culture 72 hours after explantation and 24 hours after normal feeding. Control. Unstained. $\times 75$.

FIG. 2.—$C_3H$-culture placed in a liquid medium containing 10 $\gamma$/ml. of toluidine blue immediately after explantation and fed with the same fluid after 48 hours. The photgraph was taken 72 hours after explantation. Accumulation of metachromatic toluidine blue observable in cells. Unstained. $\times 75$.

FIG. 3.—$C_3H$-culture, 72 hours after explantation and 24 hours after feeding with toluidine blue in a final concentration of 10 $\gamma$/ml. Many metachromatic granules of toluidine blue observable in cells. Unstained. $\times 75$.

1

2

3

Csaba, Ács, Horváth and Kapa.

theoretical considerations we succeeded in elaborating a method for the inhibition of tumour growth which has proved successful in animal experiments. The substances employed by us are antimetabolites rather than cytostatic substances. That this is true is shown by the fact that cytostatic substances are known to affect quickly-dividing tumour cells in the first place, while our method produces a more marked effect if cell proliferation is slow. Far from being inconvenient, this feature of our method is decidedly advantageous if we remember that—apart from haemoblastoses—tumours in human subjects are frequently slower growing than transplantable animal tumours.

### SUMMARY

Relying on the evidence of *in vitro* and *in vivo* experiments the authors conclude that tumours require heparinoid substances for their growth, and describe new possibilities for the inhibition of tumour growth. Theoretical considerations have led to the elaboration of a method by which it is possible to check the proliferation of transplantable tumour cells in about 45 per cent of the test cases. These theoretical considerations, substantiated by experimental results, open up a fresh path to new therapeutic experiments.

The authors received valuable assistance in their experiments from the staff of the United Works of Pharmaceutical Dietetic Products, Budapest, for which they want to express gratitude.

### REFERENCES

Almquist, P. O. and Lansing, E.—(1957) *Scand. J. clin. Lab. Invest.*, **9**, 179.
Asboe-Hansen, G.—(1954) 'The mast cell' in 'International Review of Cytology', Ed. Bourne and Danieli, New York (Academic Press) Vol. 3).
*Idem*, Levi, H. and Wegelius, O.—(1957) *Cancer Res.*, **17**, 792.
Cramer, W. and Simpson, W. L.—(1944) *Ibid.*, **4**, 601.
Csaba, G., Horváth, C. and Ács, Th.—(1960) *Brit. J. Cancer*, **14**, 362.
*Idem*, Törö, I. and Kiss, F. I.—(1959) *Orientacion Medica.* **8**, 1.
Kizer, D. E. and McCoy, T. A.—(1959) *Cancer Res.*, **19**, 309.
Koenig, H.—(1955) *Z. exp. Med.*, **126**, 67.
Lascane, E. F.—(1958) *Cancer*, **11**, 1110.
Rottimer, A., Levy, A. L. and Conte, A.—(1958) *Ibid.*, **11**, 351.
Shetlar, M. R., Foster, J. V., Kelly, K. H., Shetlar, C. L., Bryan, R. S. and Everett, M. R.—(1949) *Cancer Res.*, **9**, 515.
Törö, I.—(1959) Szövettenyésztés (Tissue Cultures) In A. Kovách's book: 'Methods of Examination in Experimental Medicine'. Budapest (Hungarian Publ. House of the Academy).
Weimer, H., Quinn, F. A., Redlich-Moshin, J. and Nishihara, H.—(1957) *J. nat. Cancer Inst.*, **19**, 409.
Winzler, R. J. and Smyth, J. M.—(1948) *J. clin. Invest*, **27**, 617.